# Supplementary material for: Optimizing a quantum reservoir computer for time series prediction
Source: Sci Rep. 2020 Sep 7;10:14687. doi: 10.1038/s41598-020-71673-9 (PMC7477271; doi:10.1038/s41598-020-71673-9)
Supplement: Supplementary file 1 — Supplementary Figure S1. [file 41598_2020_71673_MOESM1_ESM.pdf]

Optimizing a quantum reservoir computer for time series prediction

Aki Kutvonen[1,2,*], **Keisuke Fujii**[3], **and Takahiro Sagawa**[1]

[1]Department of Applied Physics, The University of Tokyo, 7-3-1 Hongo, Bunkyo-ku, Tokyo 113-8656, Japan
[2]COMP Center of Excellence, Department of Applied Physics, Aalto University School of Science, P.O. Box 11000, FI-00076 Aalto, Espoo, Finland
[3]Graduate School of Science, Kyoto University, Sakyo-ku, Kyoto, 606-8502, Japan
[*]aki.kutvonen@gmail.com

Supplementary material

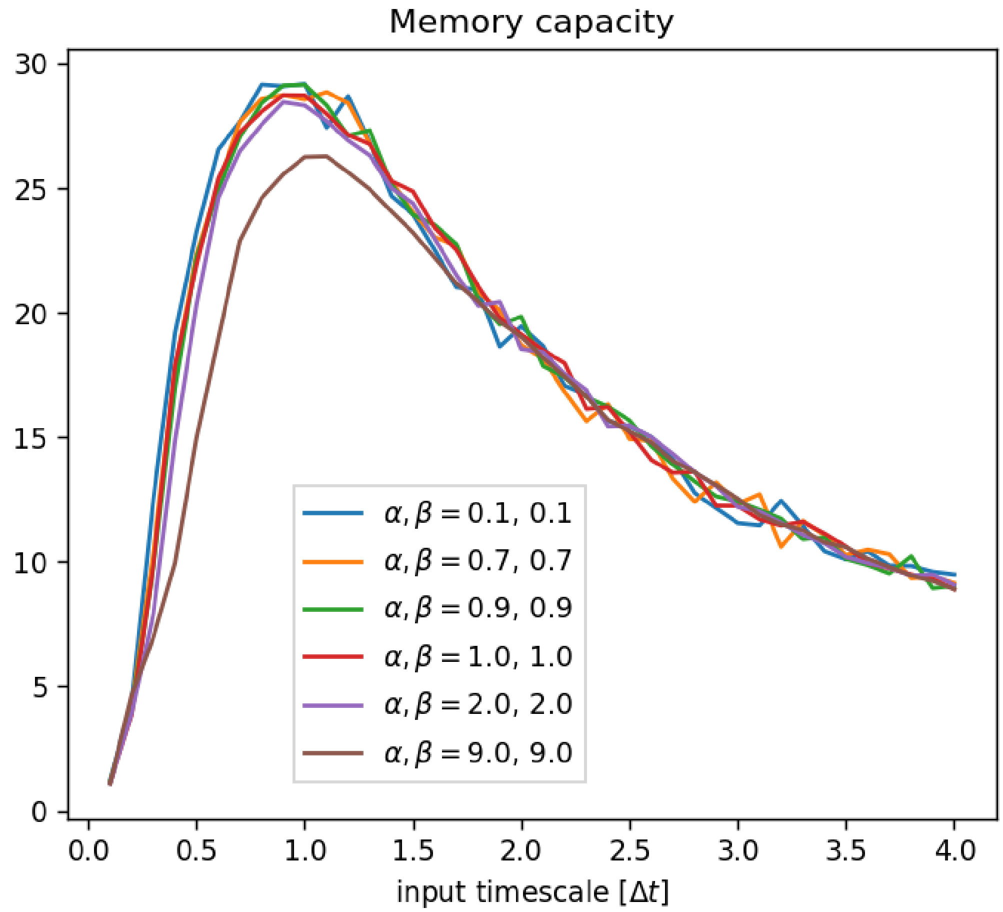

Memory capacity as a function of the input timescale Δt when the inter spin interactions J are sampled from from Beta-distribution with values $(\alpha, \beta) = \{(0.1, 0.1), (0.7, 0.7), (0.9, 0.9), (1.1), (2, 2), (9, 9), (100, 100)\}$, while keeping the value of $h = 0.5$ fixed.
